# Novel Electrospun Pullulan Fibers Incorporating Hydroxypropyl-β-Cyclodextrin: Morphology and Relation with Rheological Properties

**DOI:** 10.3390/polym12112558

**Published:** 2020-10-31

**Authors:** Deepak Poudel, Sarah Swilley-Sanchez, Sean O’keefe, John Matson, Timothy Long, Cristina Fernández-Fraguas

**Affiliations:** 1 Department of Food Science and Technology, Virginia Polytechnic Institute and State University, Blacksburg, VA 24061, USA; poudel12@vt.edu (D.P.); okeefes@vt.edu (S.O.); 2 Department of Chemistry, Virginia Polytechnic Institute and State University, Blacksburg, VA 24061, USA; sarahswilley@vt.edu (S.S.-S.); jbmatson@vt.edu (J.M.); tlong@vt.edu (T.L.); 3 Macromolecules Innovation Institute, Virginia Polytechnic Institute and State University, Blacksburg, VA 24061, USA

**Keywords:** pullulan, hydroxypropyl-β-cyclodextrin, electrospinning, rheology, entanglement concentration

## Abstract

Fibers produced by electrospinning from biocompatible, biodegradable and naturally occurring polymers have potential advantages in drug delivery and biomedical applications because of their unique functionalities. Here, electrospun submicron fibers were produced from mixtures containing an exopolysaccharide (pullulan) and a small molecule with hosting abilities, hydroxypropyl-β-cyclodextrin (HP-β-CD), thus serving as multi-functional blend. The procedure used water as sole solvent and excluded synthetic polymers. Rheological characterization was performed to evaluate the impact of HP-β-CD on pullulan entanglement concentration (C_E_); the relationship with electrospinnability and fiber morphology was investigated. Neat pullulan solutions required three times C_E_ (~20% w/v pullulan) for effective electrospinning and formation of bead-free nanofibers. HP-β-CD (30% w/v) facilitated electrospinning, leading to the production of continuous, beadless fibers (average diameters: 853-1019 nm) at lower polymer concentrations than those required in neat pullulan systems, without significantly shifting the polymer C_E_. Rheological, Differential Scanning Calorimetry (DSC) and Dynamic Light Scattering (DLS) measurements suggested that electrospinnability improvement was due to HP-β-CD assisting in pullulan entanglement, probably acting as a crosslinker. Yet, the type of association was not clearly identified. This study shows that blending pullulan with HP-β-CD offers a platform to exploit the inherent properties and advantages of both components in encapsulation applications.

## 1. Introduction

**Figure 1:**
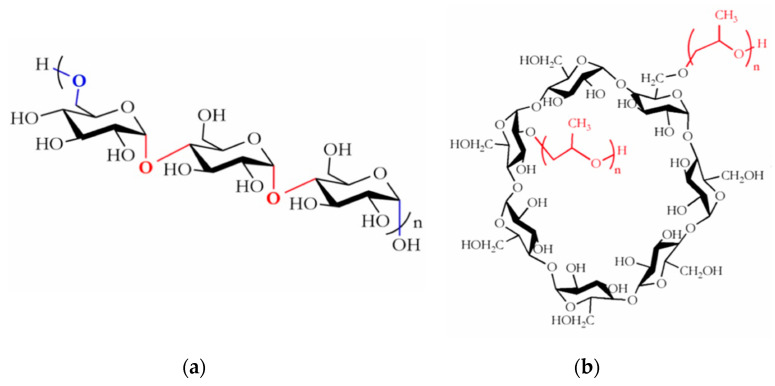
(**a**) Repeating unit of pullulan; (**b**) chemical structure of Hydroxypropyl-β-cyclodextrin (HP-β-CD).

Over the past few decades, electrospinning has drawn considerable attention in the energy, environmental, food, pharmaceutical and biomedical industries as a versatile, simple and cost-effective method to produce submicron polymer fiber mats with structural and functional advantages, under ambient conditions [1,2]. These include high surface-to-volume ratio, light weight, porous nature and flexibility for design [3,4]. Particularly, there is a growing interest in the use of electrospun fibers fabricated from natural, renewable, biocompatible and biodegradable polymers as protective packaging and carriers for bioactive compounds for a multitude of applications including smart active packaging, tissue engineering, drug delivery and/or encapsulation applications [5]. Polysaccharides are ideal substitutes for synthetic polymers derived from fossil fuels and often preferred to proteins in terms of their abundance and cost [6]. Among polysaccharides, pullulan is receiving increasing attention due to its non-toxicity, GRAS (Generally recognized as safe) label and broad regulatory acceptance, along with its unique structural properties and functionalities [7]. Pullulan (Figure 1a) is a linear, flexible and non-ionic exopolysaccharide produced by Aureobasidium pullulans in starch crops and, more recently, in waste generated from agro-industries [8]. It consists mainly of maltotriose units (whose glucose molecules are linked by α (1,4) glycosidic bonds) linked via α (1,6) glycosidic bonds, forming a stair-step type structure [9]. This distinct structural arrangement is responsible for its randomly ordered and amorphous character [10] and makes pullulan readily soluble in water, stable in aqueous solutions over a wide range of pH, non-gel forming and slightly viscous in comparison with other polysaccharides. These unique features, in turn, provide pullulan with the ability to form strong resilient electrospun nanofibers, thin layers and flexible coatings with a good oxygen barrier along with adhesive properties [9,11]. Despite pullulan being previously shown to produce ultrathin electrospun fibers in DMSO/water mixtures [12], the fabrication of pure pullulan spunfibers from aqueous solutions has been rarely explored, which is an important requisite for applications where the use of non-organic solvents is essential. Moreover, the common route to electrospinning pullulan found in the literature is either using pullulan in combination with a synthetic carrier polymer to improve electrospinnability of pullulan solutions or using pullulan as a carrier polymer for improving the solution properties of unspinnable materials. In this regard, electrospun fibers (mats) have been previously obtained from blends of pullulan and poly(ethylene) oxide [13], polyvinyl alcohol (PVA) [14], as well as natural biopolymers like pea, amaranth and whey proteins [15,16,17], chitosan [18] or gelatin [19]. A feature that, however, may exclude pullulan from some encapsulation applications is its neutral character; the lack of charge on its backbone would prevent, for example, electrostatic interactions with charged polymers. Thus, our particular approach in this work is to use cyclodextrins (CDs), non-charged, cyclic oligosaccharides with complexation properties, to functionalize pullulan electrospun fibers and widen their range of applications.

CDs, composed of different numbers of α- (1→4) glucose units, have a toroid-shaped structure (Figure 1b) that provides the molecule with a hydrophilic exterior and a protective hydrophobic internal cavity, making CDs perfect candidates not only for hosting guest molecules but also for, potentially, reorganizing polymer structures, morphologies and/or conformations [20,21]. CDs have been previously incorporated into electrospun fibers produced from petroleum-based synthetic polymers (e.g., (PVA) [22], polylacticacid (PLA) [23], poly(methyl methacrylate), polystyrene [24] and bio-based polymers like chitosan [21] and zein [25,26]). However, no studies have been done to exploit the hosting ability of CDs with the unique properties of pullulan into the formation of electrospun fiber mats using water as the sole solvent. 

Additionally, an aspect that plays a major role in determining the capacity of polymer dispersions to form fibers during electrospinning is the rheological properties of the spinning dope and the polymer entanglement concentration (C_E_) [27,28]. Therefore, this work aimed to evaluate the feasibility of fabricating electrospun fibers from aqueous blends of pullulan and HP-β-CD, a hydroxyalkyl CD derivative with improved water solubility, without the aid of any additional polymer or surfactant. Our main emphasis was to investigate the dependence of electrospinnability and spunfiber morphology on solution rheological behavior and C_E_. 

## 2. Materials and Methods 

### 2.1. Materials

Food-grade pullulan (Lot No. 8B08) was kindly provided by Hayashibara Biochemical Laboratories Inc. (Okayama, Japan), and 2-Hydroxylpropyl-β-Cyclodextrin (HP-β-CD, purity ≥ 97%, Mw ~ 1541.6 g/mol, substitution degree 5) was purchased from Cayman Chemical (Ann Arbor, MI, USA). Distilled–deionized water from an Ultrapure, Millipore Direct-Q 3 with UV water system (Hach, Loveland, CO, USA) was used in the study. 

### 2.2. Preparation of Pullulan/HP-β-CD Binary Solutions for Electrospinning 

Aqueous pullulan solutions were prepared at different concentrations (0.5, 1, 2, 5, 8, 10, 12, 15, 20 and 25% w/v) by dissolving the appropriate amount of pullulan in ultrapure water for 4 h under continuous and gentle stirring at room temperature. Pullulan/HP-β-CD blends were prepared at two different levels of HP-β-CD (10% and 30% w/v) and the above pullulan concentrations. Binary solutions were mixed at room temperature using a magnetic stirrer for at least 4 h until ensuring complete dissolution and hydration, obtaining homogeneous solutions. The samples were stored at 4 ^o^C before rheological characterization or electrospinning.

### 2.3. Rheological Characterization of Solutions for Electrospinning 

The rheological behavior of samples was characterized using a DHR3 rheometer (TA Instruments, New Castle, DE, USA) equipped with a cone plate measuring system (40 mm diameter, 2^o^ cone angle, gap 0.043 mm) and a solvent trap to minimize moisture loss. The test temperature was set at 25 °C by using a circulating water system. The viscoelastic behavior of polymer solutions and blends was studied by performing small-amplitude oscillatory shear (SAOS) measurements. First, amplitude tests were performed at strains ranging from 0.01% to 100% and at fixed angular frequency (ω) of 1 Hz to define the linear viscoelastic region (LVE). Frequency sweeps were conducted from 0.1 to 100 rad/s at a constant shear strain within the LVE region to determine the evolution of the elastic (G’) and viscous (G”) modulus as a function of oscillation frequency (ω). 

Flow curves were obtained by recording apparent viscosity at shear rates ranging from 0.01 to 600 s^−1^. Data were fitted to the power law model (Equation (1)):

(1) $$\eta \;=\;k\;{\left(\dot{\gamma }\right)}^{n-1}$$

where η is the apparent viscosity (Pa s), $\dot{\gamma }\;$is the shear rate (s^−1^), k is the consistency index (Pa s^n^) and n is the flow behavior index. 

Specific viscosity (η_sp_) was calculated by using the following equation:

η_sp_ = (η_o_ − η_s_)/η_s_ (2)

where η_s_ is the viscosity of the solvent and η_0_ is the zero-shear viscosity, which correspond to the actual or extrapolated values of apparent viscosity at 0.1 s^−1^ from the flow curves. Specific viscosity data were plotted against the polymer concentration in order to determine the entanglement concentration (C_E_).

### 2.4. Dynamic Light Scattering Measurements 

Dynamic light scattering (DLS) measurements were performed using a Zetasizer Nano ZS (Malvern Instruments, U.K.) at 25 °C. Aqueous solutions containing pure pullulan or HP-β-CD and the pullulan/HP-β-CD mixture were filtered and analyzed via DLS. The refractive index of pullulan and HP-β-CD used was 1.6 and 1.59, respectively.

### 2.5. Electrical Conductivity Measurements

The electrical conductivity of Pullulan solutions and blends with HP-β-CD was measured using a conductometer (Digital EC Meter DEC-2, ATAGO, Japan) at room temperature.

### 2.6. Fabrication of Electrospun Fibers 

Pure pullulan solutions and pullulan/ HP-β-CD blends were placed in a 5mL syringe fixed to a syringe pump (KD Scientific Inc, New Hope, PA, USA). The positive lead of a high-voltage power supply (Spellman CZE1000R; Spellman High Voltage Electronics Corp., Hauppauge, NY, USA) was connected to the 18-gauge syringe needle via an alligator clip. A grounded metal static collector plate (304 stainless steel mesh screen) covered by aluminum foil was placed 15 cm from the needle tip. The syringe pump delivered the solution at a controlled flow rate of 0.5 mL/h, and the voltage was maintained at 15 kV. Electrospinning was performed at room temperature (~20 °C) and ambient humidity (~50%) 

### 2.7. Characterization of Electrospun Nanofibers 

#### 2.7.1. Morphological Analysis 

Electrospun fiber morphology was studied using a Field-Emission Scanning Electron Microscope (FESEM) (LEO 1550, Zeiss, Oberkochen, Germany) and an accelerating voltage of 5 kV. Fibers were collected on a 1⁄4 × 1⁄4 in. stainless steel mesh, mounted on a SEM disk and sputter-coated with a 10 nm Pt/Au layer to reduce electron charging effects. In order to determine fiber size, at least 100 different fibers from each sample were randomly selected from SEM images and their diameter histograms were obtained by using Image J software (Bethesda, Rockville, MD, USA).

#### 2.7.2. Thermal Analysis 

The thermal properties of both spunfibers and physical mixtures of individual components (i.e., pullulan and HP-β-CD powders) were examined by using a Differential Scanning Calorimeter (DSC) (Q-2000, TA Instruments, Newcastle, DE, USA) equipped with a nitrogen cooler system. The pure component powders were mixed in a mortar and sieved through a 100-mm mesh sieve. A specific amount of sample (4 ± 0.8 mg) was directly weighed in an aluminum pan, hermetically sealed and heated from 25 to 250 °C at a heating rate of 10 °C/min. The instrument was equilibrated for at least 60 min before starting scanning, and an empty pan was used as reference. Indium standard 2.7 was used for calibration. The magnitude of the enthalpy change (∆H) associated with thermal transitions was calculated from the peak area using the Universal Analysis software (TA Instruments, New Castle, DE, USA).

#### 2.7.3. Fourier Transform Infrared Spectroscopy (FTIR) Analysis 

Infrared spectra of the electrospun fiber mats were obtained with a ThermoFisher Scientific Nicolet iS5 fourier transform infrared spectrometer with an iD7 ATR (Attenuated Total Reflection) accessory equipped with a diamond cell in attenuated total reflection mode. Pullulan and HP-β-CD spectra were collected with powder standards; their physical mixture (prepared by dissolving pullulan and HP-β-CD in water and allowing the solution to dry) was also analyzed. Pullulan and HPBCD spectra were collected with powder standards, and the pullulan/HP-β-CD physical mixture was prepared by dissolving pullulan and HPBCD in water and allowing the solution to dry. Samples were scanned at operating wavelengths in the range between 4000 and 600 cm^−1^ with 1 cm^−1^ wavenumber resolution, and each measurement consisted of 50 scans at room temperature.

### 2.8. Statistical Analysis 

All measurements were performed in triplicate and values are reported as means and standard deviation. The data were analyzed by conducting an analysis of variance (ANOVA). Tukey’s multiple comparison test was conducted to evaluate significant differences among experimental mean values using Prism (version 8, GraphPad Software, Inc., La Jolla, CA, USA). The level of significance was set at *p* < 0.05.

## 3. Results and Discussion

### 3.1. Rheological Properties of Electrospinning Solutions 

#### 3.1.1. Viscoelastic Behavior 

**Figure 2:**
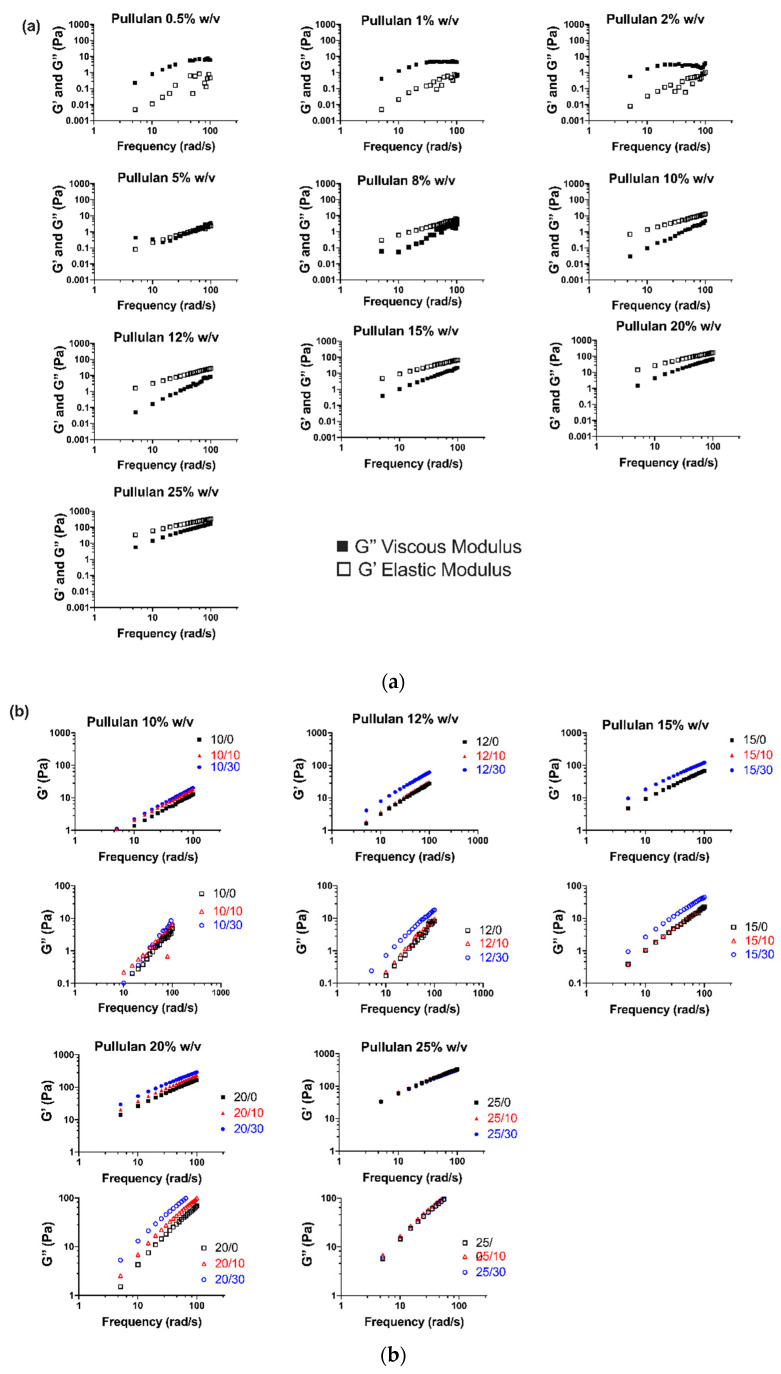
(**a**) Mechanical spectra of pullulan solutions at concentrations ranging 0.5–25% (w/v) showing the variation of elastic modulus, G’ (open symbols), and viscous modulus, G” (filled symbols), with frequency. (**b**) Effect of HP-β-CD (10 and 30% w/v) addition to pullulan solutions on the elastic modulus, G’, and viscous modulus, G”.

In order to investigate the effect of HP-β-CD on the viscoelastic behavior of aqueous pullulan solutions, amplitude (not shown) and frequency sweep oscillatory tests were performed (Figure 2). Figure 2a shows the evolution of the elastic (G’) and viscous (G’’) moduli of neat pullulan solutions at different concentrations (0.5–25% w/v) with angular frequency. Increasing the concentration of pullulan resulted in higher G’ and G’’, and at a fixed pullulan concentration, both moduli values increased with frequency. Pullulan solutions at concentrations below 5% (w/v) behaved as viscoelastic liquids, as shown by the greater G’’ values compared to G’ during the whole range of frequency. Overlap between elastic and viscous moduli curves occurred at pullulan 5% (w/v), resulting in G’ > G’’ at concentrations above 5% (w/v), which reflects the predominantly solid-like viscoelastic character of these solutions; this behavior is characteristic of concentrated polymer solutions. The larger gap at G’>G’’ observed at low frequencies might imply that pullulan chains start to form an entangled network; nonetheless, the higher dependence of G’’ on the frequency compared to G’ shows that the response of pullulan at 8–15% (w/v) is still somewhat influenced by viscous behavior. At pullulan 20–25% (w/v), the gap between G′ and G′′ diminished at lower frequencies, which might be attributed to the formation of a still weak but more entangled network. The addition of HP-β-CD increased the viscoelastic character of pullulan, with higher G’ and G’’ values for 10–20% (w/v) pullulan solutions, the effect being more significant with the incorporation of HP-β-CD 30% w/v (Figure 2b). 

This enhanced viscoelasticity could be due to non-covalent associations (i.e., Van der Waals forces, hydrogen bonds) of HP-β-CD’s hydroxyl-propyl groups with pullulan, which could create weak networks between polymer chains, thus somewhat improving G’ and G’’ performance [29]. However, at higher pullulan concentrations, HP-β-CD had no effect on pullulan viscoelasticity, i.e., G’ and G’’ of pullulan 25% (w/v) did not change with HP-β-CD addition. These results suggest that HP-β-CD 30% w/v might be able to promote crosslinking of pullulan chains at maximum pullulan concentrations of 20% (w/v), assisting in polymer entanglement.

#### 3.1.2. Flow Behavior 

**Figure 3:**
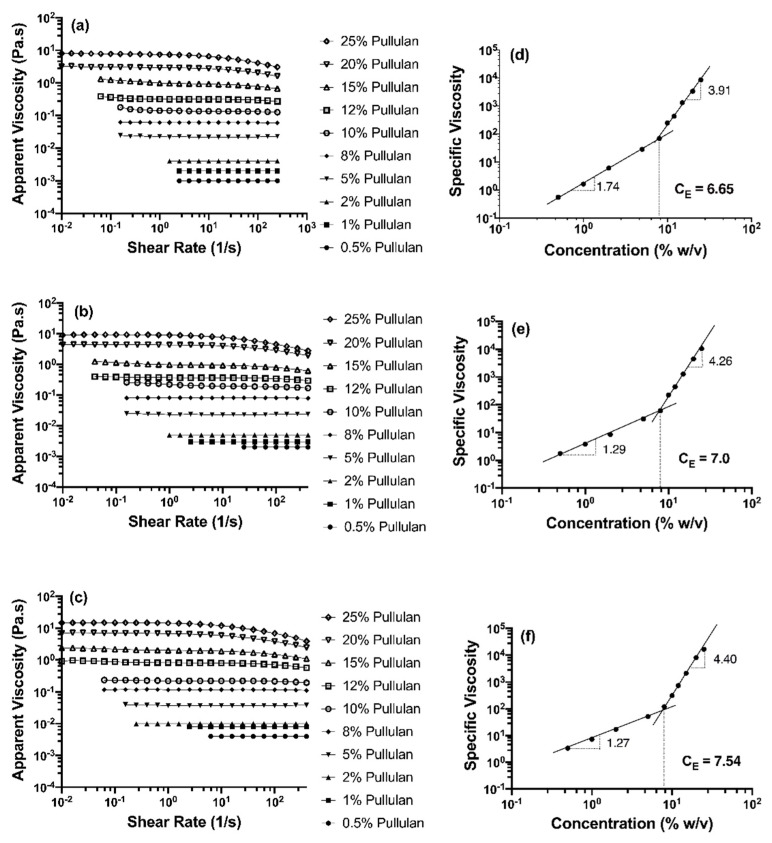
Flow curves of pullulan aqueous solutions at different concentrations (0.5–25% (w/v)) added with (**a**) 0, (**b**) 10 and (**c**) 30% (w/v) HP-β-CD. Plots of specific viscosity versus pullulan concentration for blends with (**d**) 0, (**e**) 10 and (**f**) 30% (w/v) HP-β-CD. The slopes of fitted lines (scaling exponents) in the semi-dilute entangled and semi-dilute unentangled regions, as well as the entanglement concentrations, C_E_ (indicated with a vertical line), are illustrated.

Figure 3 shows the flow curves of aqueous pure pullulan solutions and their blends with HP-β-CD (10 and 30% w/v), and their respective entanglement concentration (C_E_) plots. Power law parameters were calculated using Equation (1), and the flow behavior index (n) and consistency index (k) of samples are shown in Table 1. Pullulan solutions with or without added HP-β-CD obeyed the power law (r^2^ ≈ 0.99). As shown in Figure 3a, pure pullulan solutions at concentrations of 8% (w/v) or lower showed Newtonian behavior (i.e., shear viscosity was constant with shear rate), whereas solutions at concentrations above 8% (w/v) could be defined as non-Newtonian. The viscosity of pullulan solutions >8% decreased very slowly with increasing shear rate until 200 s^−1^, indicating a very weak shear thinning behavior. Similarly, a previous study reported that solutions of pullulan in DMSO as a solvent showed shear thinning behavior at pullulan concentration ≥10% (w/v) [12].

As the concentration of pullulan increased, n values decreased, confirming a shift from Newtonian to non-Newtonian behavior. In accordance with the profile of the flow curves, while pullulan solutions < 8% (w/v) displayed n values ~1, concentrations ≥ 8% (w/v) led to n values lower than 1 (ranging from to 0.978 to 0.692 for 8 and 25% (w/v) pullulan), confirming more pronounced, but still weak, shear-thinning behavior only at high shear rates, with increasing pullulan concentration. A similar flow profile and slightly shear thinning behavior, but with greater apparent viscosity, was observed when incorporating HP-β-CD (Figure 3b,c). As HP-β-CD concentration increased, the increase in k values and decrease in n values of pullulan were more significant; for example, at 20% (w/v) pullulan, k values increased from 6170 to 8925 and 16,392 mPa.s^n^ and n values decreased from 0.754 to 0.746 and 0.695, when HP-β-CD increased from 10 to 30% (w/v) (Table 1). This indicates that HP-β-CD slightly enhanced the shear thinning behavior of pullulan. This could be due to the enhanced formation of interpolymer chain networks, which led to an enhancement in the viscosity with the incorporation of higher amounts of HP-β-CD [30]. In contrast, the decrease in viscosity reported for more hydrophobic polymers with the addition of cyclodextrins [24] has been attributed to the decoupling of polymer associations caused by the complexation between the CD and the polymer hydrophobic moieties [30].

#### 3.1.3. Entanglement Polymer Concentration (C_E_) 

Molecular entanglement of polymer chains is an important prerequisite for the spinnability of polymer solutions and formation of electrospun fibers [31]. The concentration at which polymer entanglement (C_E_) occurs is described as the boundary between the semi-dilute unentangled and entangled regimes where a significant overlap of the polymer chains topologically constrains the chain motion [32]. In the semi-dilute unentangled region, the polymer chains overlap one another without entanglement occurring, while in the semi-dilute entangled region, the chains considerably overlap. Since the effect of chain entanglement probably positively correlates to viscosity, from the flow curves, we can identify C_E_ and predict the minimum concentration of polymer required for the formation of electrospun fibers. Figure 3d–f shows specific viscosity (η_sp_) data vs. pullulan concentration (% w/v), scaling exponents (i.e., plot slopes) and C_E_ values for neat pullulan solutions and pullulan-HP-β-CD blends. C_E_ was determined from the intercept of the fitted lines in the semi-dilute unentangled and entangled regions [12]. The C_E_ of aqueous neat pullulan solutions was found to be 6.65% (w/v), and the scaling exponents of neat pullulan solutions were c ^1.74^ and c ^3.91^ in the semi-dilute unentangled and entangled regime, respectively (Figure 3d). For neutral, linear polymers (like pullulan) in a good solvent, η_sp_ is proportional to c ^1.25^ in the semi-dilute unentangled regime, while η_sp_ ∼ c ^4.8^ in the semi-dilute entangled regime [33]. The semi-dilute untangled regime for pullulan (η_sp_ ∼ c ^1.74^) in water was greater than the theoretical predictions (η_sp_ ∼ c ^1.25^) and the reported values of pullulan in DMSO (η_sp_ ∼ c ^1.39^) [12] as well as for linear and branched poly(PET-co-PEI) in chloroform/dimethylformamide (η_sp_ ~ c ^1.41^) and (η_sp_ ~ c ^1.39^), respectively [27]. The greater concentration dependence of η_sp_ observed in aqueous pullulan solutions indicates that the type of solvent affects molecular interactions between pullulan and suggests a more linear and extended conformation of pullulan in water. In the semi-dilute entangled regime, the concentration dependence of η_sp_ for pullulan in water was c ^3.91^. This value is lower than the theoretical prediction for linear polymers (η_sp_ ∼ c ^4.8^) and similar to that of other random-coil polysaccharides, such as dextran and alginate [34], suggesting that despite pullulan molecules being entangled with each other, they were also associated with water, which probably prevents a strong interchain interaction. When incorporating 10 and 30% w/v HP-β-CD, pullulan’s C_E_ was not significantly shifted (Figure 2e, f). However, the scaling exponent in the semi-dilute unentangled region significantly decreased from c ^1.74^ to c ^1.28^ and to c ^1.26^, respectively, which was in accordance with the theoretical prediction of linear polymers in a good solvent (η_sp_ ∼ c ^1.25^) and suggests a change in pullulan chain conformation as a result of the presence of HP-β-CD and that the molecule does not diminish pullulan interactions. In the semi-dilute entangled regime, there was an increase in the exponent from c ^3.91^ to c ^4.26^ and c ^4.40^ for mixtures incorporating 10 and 30% (w/v) HP-β-CD, respectively, being significant for 30% (w/v) HP-β-CD. This rise in slope, even if it was not substantial, suggests that HP-β-CD promotes in some way intermolecular associations between pullulan chains [21,27]. A greater increase in scaling exponents (c ^3.8^ to c ^7.2^) has been observed when 50% (w/v) HP-β-CD was added to chitosan in 10% acetic acid solutions [21]. The divergence from our data is due to the greater HP-β-CD concentration used, the cationic and more hydrophobic nature of chitosan and its specific behavior in the solvent. A similar increase in scaling exponents to that for chitosan (c ^4.5^ to c ^8^) was observed in poly(alkyl methacrylate) modified with additional hydrogen bonding groups that improve interchain interactions [27]. Therefore, it would be reasonable to suggest that 30% HP-β-CD assists in the entanglement of (adjacent) pullulan chains, probably acting as a crosslinker, even if it does not promote a strong interchain interaction, as seen by the lower increase in scaling exponent.

### 3.2. Electrospinning of Pullulan-HP-β-CD Fibers 

The electrospinning of pullulan/HP-β-CD fibers was carried out by using water as a solvent since HP-β-CD is soluble in water; hence, we were able to prepare homogeneous pullulan/HP-β-CD solutions that did not form two separate aqueous phases. Accordingly, pullulan and its blends with HP-β-CD were electrospun from aqueous solutions at 15 kV, 3 mL/h flow rate and 15 cm distance from the syringe tip to the grounded target. These electrospinning conditions were optimized by performing a series of preliminary experiments testing various electric voltages, flow rates and tip-collector distances. Pullulan electrospun fibers were collected as fibrous mats, characterized in terms of morphological and thermal properties and analyzed by FTIR. Since the process parameters were kept constant during the experiments, it is expected that variations in fiber morphology and size are mainly due to changes in the spinning solution properties.

#### 3.2.1. Electrospun Fiber Morphology and Correlation with C_E_

**Figure 4:**
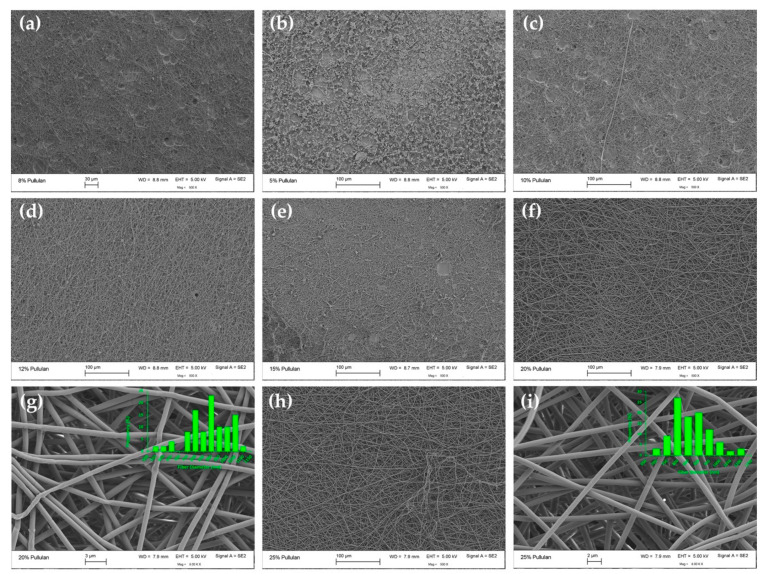
SEM images and diameter histograms of pullulan electrospun fibers at (**a**) 5, (**b**) 8, (**c**) 10, (**d**) 12, (**e**) 15, (**f**,**g**) 20 and (**h**,**i**) 25% (w/v). Fiber size distributions are only presented for smooth and bead-free spunfibers.

**Figure 5:**
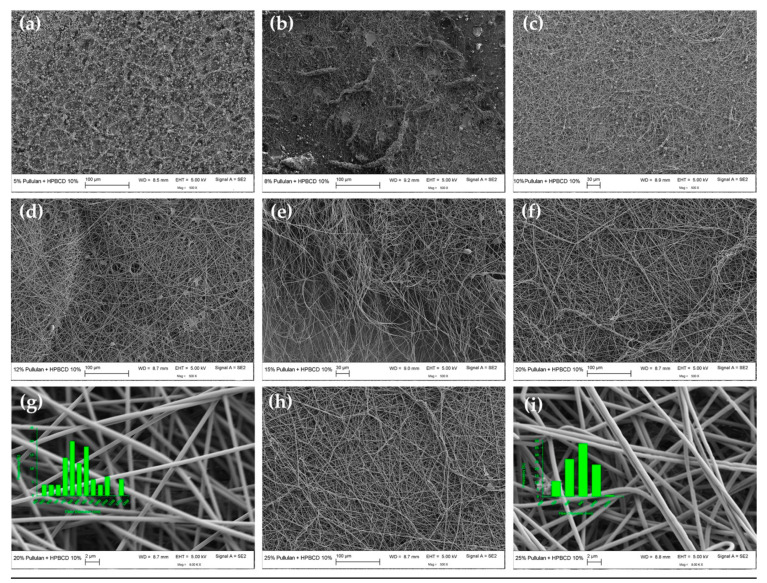
SEM images and diameter histograms of electrospun fibers containing 10% (w/v) HP-β-CD and different pullulan contents: (**a**) 5, (**b**) 8, (**c**) 10, (**d**) 12, (**e**) 15, (**f**,**g**) 20 and (**h**,**i**) 25% (w/v). Fiber size distributions are only presented for smooth and bead-free spunfibers.

**Figure 6:**
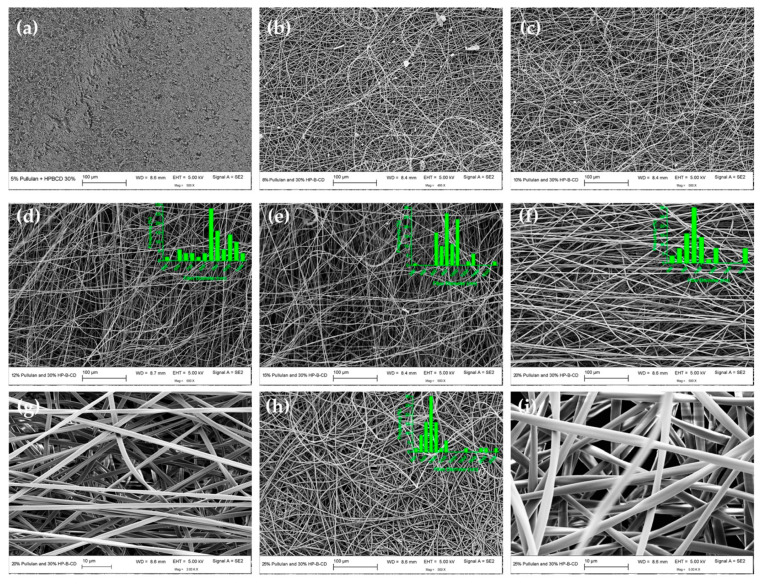
SEM images and diameter histograms of electrospun fibers containing 30% (w/v) HP-β-CD and different pullulan contents: (**a**) 5, (**b**) 8, (**c**) 10, (**d**) 12, (**e**) 15, (**f**,**g**) 20 and (**h**,**i**) 25% (w/v). Fiber size distributions are only presented for smooth and bead-free spunfibers.

Visual observation and SEM were used to evaluate the morphology of electrospun fibers. Representative SEM images and fiber size distributions of electrospun fibers produced from pure pullulan solutions and from binary mixtures containing 10 or 30% of HP-β-CD are shown in Figure 4, Figure 5 and Figure 6, respectively. The morphology and average diameters of the spunfibers are summarized in Table 1. Attempts at electrospinning 10 and 30% w/v aqueous solutions of pure HP-β-CD (levels tested in the blends) were unsuccessful, yielding no fibers, and only droplets were observed (images not shown). Increasing the HP-β-CD concentration to 150% (w/v) led to the creation of non-homogeneous spunfibers but there were plenty of beads and defects (Supplementary Figure S1). Celebioglu and Uyar reported the formation of uniform HP-β-CD nanofibers with some elongated beaded structures from 140% (w/v) HP-β-CD solutions in water [35]. The small divergence from our results might be due to the greater substitution degree of the HP-β-CD used in our study, around 5 versus 0.6-0.9, which probably affected the viscosity of the resulting solutions. Thus, the degree of substitution could be an important parameter to consider when attempting to electrospin pure HP-β-CD and their blends with polymers. 

Figure 4 shows that spunfiber morphology depended on the concentration of the solution from which neat pullulan was electrospun. At concentrations below the C_E_ (6.65%, w/v), electrospinning was not smooth and was interrupted by electrospraying or a Taylor cone did not form due to the low viscosity (Figure 3a) and low conductivity (Supplementary Table S1) of the spinning dope, resulting mostly in bead formation (Figure 4a). At low viscosities, the large amount of solvent molecules and few chain entanglements make the surface tension the dominant factor on the electrospinning jet, causing bead formation [36]. As the pullulan concentration was increased beyond the C_E_ and below <15% (w/v), the greater level of chain overlap led to constraint of the chains and the formation of entanglement couplings; jet formation was initiated and thus heterogeneous mats composed by beads, beaded fibers and fibers were generated (Figure 4b–e). 

The amount of beads decreased and the beads’ shapes became more elongated with increasing polymer concentration. At pullulan concentrations 3 and 3.75 times the C_E_ (i.e., 20 and 25% (w/v) respectively), the jet formed a Taylor cone and bead-free fibers with average diameters ~863–886 nm (Table 1, Supplementary Figure S2) were collected as fibrous membranes (Figure 4f–i). As these images indicate, 20% (w/v) is the optimal lowest pullulan concentration for generating uniform, defect-free neat pullulan spunfibers at the applied electrospinning conditions from aqueous solution. Increasing polymer concentration derived in the presence of more polymer chain entanglements that increased solution viscosity allowed the electrified polymer jet to be fully stretched, hence eliminating the beaded structures [13,37,38]. This behavior is characteristic of many polymer–solvent systems during electrospinning. The increase in conductivity could have also caused greater mobility of ions, contributing to the elongation of the polymer jet and thus to the reduction in the amount of beads and beaded fibers observed as the pullulan concentration increased. Yet, the conductivity of 20 and 25% (w/v) pullulan solutions was still low, which, together with the viscosity of these solutions, could explain the relatively large diameters of the collected pullulan fibers (~863–886 nm). Previously reported diameters of pullulan spunfibers from wet-electrospinning and dry-electrospinning are in the range ~300 nm–15 μm [12] and 50–700 nm [39,40,41], respectively, which in some cases no longer belong to the nano-range. The divergence of diameters is due to the different solvents used and electrospinning process variables. Since water was used as a solvent in our study, the high surface tension of the aqueous solutions might be another factor responsible for the obtained fiber sizes [42]. Our results correlate with previous studies showing the formation of uniform nanofibers from pure solutions of pullulan at concentrations of 15% (w/v) and beyond when water was used as a solvent [40]. 

The effect of HP-β-CD on the morphology of electrospun pullulan fibers was dependent on the HP-β-CD level (Figure 5 and Figure 6, Table 1). 

Blends of pullulan and 10% HP-β-CD showed similar behavior over the same concentration regimes as neat pullulan; however, HP-β-CD created a more viscous blend of similar conductivity that was somewhat more spinnable (i.e., better defined Taylor cone, quicker occurrence of fiber deposits on the collector screen and formation of a mat that was more easily removed from the plate and handled) than pullulan alone and assisted in reducing the number of defects, like beads, without increasing polymer concentration (Figure 5). For example, with the addition of 10% HP-β-CD to pullulan 15% w/v, the morphology transitioned from multiple beaded fibers (Figure 4e) to smoother fibers with only a few beads (Figure 5e). Yet, although the spunfiber diameter decreased, it was not significantly affected (Table 1, Figure S2). The decreasing trend in the mean diameter from 886 to 863 nm with increasing pullulan concentration from 20 to 25% (w/v), and from 853 to 823 nm with the incorporation of 10% HP-β-CD in these solutions, was not expected as solutions of higher viscosity with similar conductivity would yield thicker fibers. The diameter reduction (although it was not significant) may be due to a potential secondary jet erupting from the main electrospinning jet which was stable enough to yield thinner fibers at a certain viscosity [43]. The humidity of the spinning environment (~50%) could have played a role in this phenomenon as increased humidity leads to an increase in the conductivity of the medium, which, apart from allowing more jet stretching, could form secondary jets and split the fibers [44]. This may also explain the wide fiber diameter distribution and some defects observed in the microstructure of the nanofibers (e.g., fiber bundles). 

When raising HP-β-CD concentration to 30% (w/v) (Figure 6), a more significant improvement of electrospinnability was observed, which translated into an enhancement in pullulan fiber morphology. The diameter of pullulan spunfibers was marginally affected by increasing the HP-β-CD content from 10 to 30% (w/v) (Table 1, Figure S2). 

**Figure 7:**
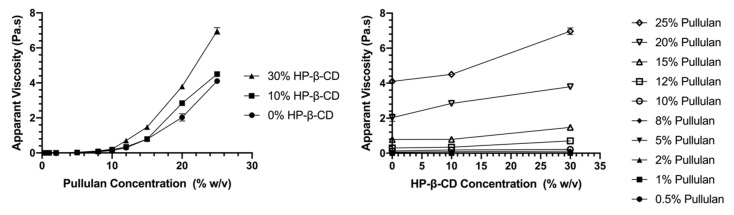
Plot of apparent viscosity (at shear rate of 100 s-1) versus (**a**) pullulan concentration (% w/v) and (**b**) HP-β-CD concentration (% w/v).

As seen in Figure 6, when blends of pullulan and 30% HP-β-CD were electrospun from solutions below the C_E_ (7.54% (w/v)), droplets and beads were mostly formed (Figure 6a). As the concentration increased beyond the C_E_—for example, at 12% (w/v) (1.59 times C_E_)—bead-free nanofibers were formed with average diameters of 866 nm (Figure 6d). At concentrations two times C_E_ (i.e., 15% (w/v)), uniform, defect-free fibers with nominal diameters of 886 nm were formed (Figure 6e). As the concentration was further increased to 20 and 25% (w/v), uniform spunfibers with average diameters of 882 and 1019 nm were respectively spun (Figure 6f–i). These results indicate that the incorporation of 30% HP-β-CD allows for the formation of continuous and smooth, bead-free pullulan spunfibers of slightly larger diameter, at lower polymer concentrations than those required in the neat pullulan systems. This improvement in electrospinnability and fiber morphology could be explained by the increased conductivity (at low pullulan concentrations) and the more pronounced rise in solution viscosity observed for pullulan solutions, mainly at concentrations ~12-25% w/v, with the incorporation of 30% HP-β-CD (Figure 7, Table 1). As the solution viscosity increased, a consequence of the increased number of entanglement couplings that act as sites for the crosslinking of the polymer chain, the electrified jet was fully stretched during electrospinning, thereby yielding defect-free fibers. Therefore, despite the fact that the addition of HP-β-CD did not significantly affect the polymer C_E_, the HP-β-CD inclusion increased the scaling exponent after the polymer C_E_, suggesting that HP-β-CD facilitated pullulan chain association and/or entanglement [21] as previously discussed (Figure 3f). Other studies have shown similar enhancement in electrospun fiber formation of different polymers and polysaccharides, such as polystyrene, polyethylene oxide (PEO) and zein, with the addition of CDs [13,25,45]. The larger number of entanglements might also result in electrospun fibers with larger average diameters as the concentration of HP-β-CD increased from 10 to 30% (w/v) (Table 1, Figure S2). In addition, it has been previously reported that HP-β-CD molecules form aggregates, which increase in number and size at higher concentrations and lead to increased diameters [46]. DLS measurements showed that HP-β-CD self-aggregates at 30% (w/v) (Supplementary Figure S3). Thus, the specially thick fibers collected at 25% pullulan/30% HP-β-CD (1019 ± 200 nm), the thickest among the fabricated spunfibers, could be attributed to the greater level of entanglement and significantly greater viscosity of this solution, and the presence of a higher amount of HP-β-CD aggregates. Previous studies evaluating the impact of cyclodextrins on the diameter of poly (lactic acid) fibers found increased fiber diameters with increasing β-CD concentration, even though the β-CD concentration did not proportionally increase the mean diameter [23]. Likewise, in our study, the overall trend of the diameter falling first and then rising with HP-β-CD addition and total solution concentration (Figure S2) may be due to the different roles played by conductivity and viscosity on fiber size. Additionally, the lack of tight control of environmental conditions (i.e., humidity) might be an important factor responsible for the broad distribution and large standard deviation of fiber diameters.

Based on the complexation properties of CDs, we could hypothesize that improved pullulan association/entanglement due to HP-β-CD inclusion might be due to either non-covalent interactions or complexation between pullulan and HP-β-CD. Due to the hydrophilic character of pullulan, we did not anticipate complexation of pullulan within the hydrophobic interior of HP-β-CD’s cavity. We have observed that the presence of pullulan within HP-β-CD solutions does not impede complexation between quercetin and HP-β-CD (Deepak et al., manuscript in preparation), suggesting that pullulan might not be encapsulated (at least completely) into or shield the cavity of HP-β-CD. We examined the assembly of pullulan and HP-β-CD (Figure S3) and observed a bimodal distribution for pure pullulan solutions, with the second peak being the most abundant. Likewise, HP-β-CD displayed a bimodal distribution but the first peak was the most abundant in this case. In contrast, the blend displayed a single peak with an average size between the first and second peak of pure components, which supports the self-assembling between pullulan chains and HP-β-CD’s hydroxyl-propyl groups, which may be acting as branch chains. This is in good agreement with the enhancement of viscoelastic moduli (Figure 2b) and viscosity data (Figure 7) as higher values were recorded for the blends.

#### 3.2.2. Thermal Characterization of Spunfibers 

**Figure 8:**
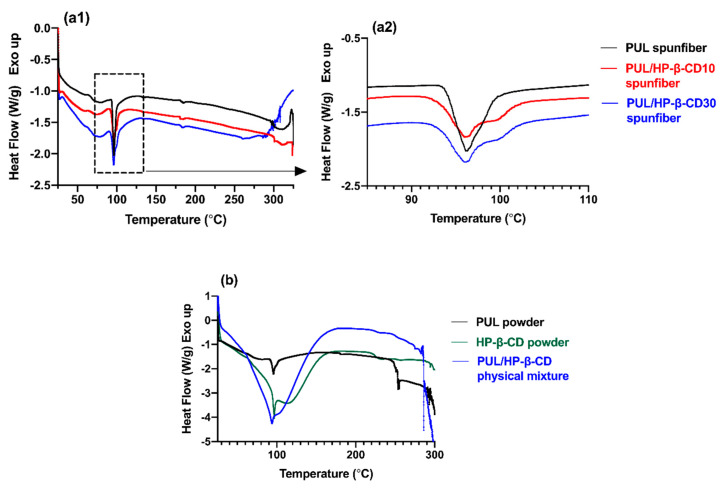
(**a1**) Differential Scanning Calorimetry (DSC) thermograms and (**a2**) enlarged region of thermograms between 85 and 110 ◦C of pullulan electrospun nanofibers containing 0% (black), 10% (red) and 30% (blue) of HP-β-CD. Polymer solution concentration 20% (w/v). (**b**) DSC thermograms of pure ground components (pullulan, HP-β-CD) and pullulan/HP-β-CD physical mixture.

Differential scanning calorimetry (DSC) is a convenient technique to investigate the compatibility of polymer blends and gives information about potential interactions between components in a matrix [47,48]. Thus, in addition to studying potential interactions between pullulan and HP-β-CD occurring within the solution, we also studied the thermal characteristics of electrospun fibers and their physical mixtures. Figure 8 shows the DSC thermograms of pullulan/HP-β-CD electrospun fibers at a polymer concentration of 20% (w/v) (Figure 8a), as well as the DSC profiles of pure components (powders) and their physical mixture (Figure 8b). Pullulan and pullulan/HP-β-CD blends exhibited two endothermic events before and after electrospinning. The magnitude of the enthalpy change (∆H) associated with endothermic transitions was calculated from the peak area. For electrospun pullulan fibers (Figure 8a) and pullulan powder (Figure 8b), the first transition, observed between 92 and 105 °C with peak temperature at 96.18 °C and ΔH of 2.921 J/g, is probably a result of the elimination of combined water. Conflicting results have been reported for the interpretation of this thermal event; previous studies state that, since pullulan is amorphous in nature, it shows a range of melting temperatures, with endothermic transitions instead of a clear melting point [49,50]. These authors associated this first peak with the loss of moisture from pullulan either in the form of spunfibers or as ground powder. However, Karim et al., who studied the thermal properties of pullulan/montmorillonite and pullulan/PVA nanofibers, attributed the endothermic peaks found at (95–99 °C) in the thermograms of pure pullulan spunfibers to a melting transition temperature (T_m_) [40,51]. The second endothermic event, observed in the vicinity of 300 °C, corresponds to the decomposition temperature of pullulan thermal degradation. The thermogram of pullulan or any of the spunfibers showed no evident glass transition under the DSC experimental conditions tested, in agreement with previous studies [52,53]. The inclusion of HP-β-CD in the pullulan spunfibers did not significantly shift the temperature at which the first endothermic event occurred, but the peak gradually broadened and shortened and showed decreased areas (Figure 8a2), suggesting that the retention of water by the spunfibers was reduced. This decreased interaction between pullulan and water when HP-β-CD was present could be due to the increased number and strength of crosslinkages between the polymer matrix and the CD which prevented pullulan from combining with water. Regarding the second endothermic event, these results suggest that the addition of HP-β-CD to pullulan slightly increases the thermal stability of PUL/ HP-β-CD blend spunfibers. 

When comparing the thermograms of bulk pullulan and HP-β-CD, i.e., as received powders, and their physical mixture (Figure 8b), we can observe a different pattern to that displayed by the spunfibers. The thermogram of HP-β-CD shows a broad and large endothermic peak at 95.12 ^o^C (∆H = 142.9 J/g) which is attributed to the elimination of water molecules bound to HP-β-CD, as previously reported in previous DSC studies on HP-β-CD [54]. There was another endothermic event at ~315 °C characteristic of HP-β-CD thermal degradation [54]. No Tg is observed as HP-β-CD is not crystalline; while most cyclodextrins are crystalline, HP-β-CD is not [21]. The pullulan/HP-β-CD physical mixture shows a broad peak at 93.54 °C and ∆H=97.26 J/g, and slightly higher thermal stability than its individual components. However, a very different profile was observed in the thermograms of pullulan/HP-β-CD spunfibers, which were coincident with that of pullulan (endothermic peak at 95.12 °C, ∆H=1.716 J/g). In addition, the broad and large endothermic peak originally observed in the HP-β-CD thermogram disappears in the pullulan/HP-β-CD spunfibers (∆H = 95.54 J/g decreased to ∆H = 2.731 J/g and 2.803 J/g in pullulan/HP-β-CD spunfibers), suggesting the existence of a more intimate association between pullulan and HP-β-CD in the spunfiber. 

#### 3.2.3. Fourier Transform Infrared Spectroscopy (FTIR) Analysis 

**Figure 9:**
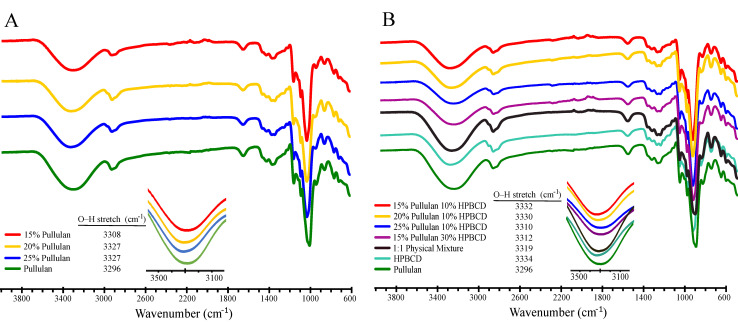
FTIR spectra of (**A**) pure pullulan and pullulan electrospun fibers, and (**B**) pure pullulan, HP-β-CD and pullulan/HP-β-CD physical mixture, and electrospun fibers of varying pullulan: HP-β-CD content. Traces have been normalized to the intensity of their strongest peak and shifted vertically for clarity. An inset is included to highlight peak shifts for the hydroxyl groups (O–H) stretches.

ATR-FTIR spectroscopy was used to further understand interactions between pullulan and HP-β-CD during spunfiber formation. FTIR spectra of pullulan (pure ground) and pullulan electrospun fibers are shown in Figure 9A. An inset is included to highlight peak shifts for the hydroxyl group (O–H) stretches, which are represented by the bands at ~3300 cm^−1^. The band at ~2900 cm^−1^ is assigned to the C-H stretch, whereas the band at ~1000 cm^−1^ corresponds to the C-O stretch. Characteristic peak values of O–H and C-O stretches are shown also in Table S2. Due to the resolution of the FTIR instrument used, we note that shifts in wavenumbers of <10 cm^−1^ are insignificant, so further discussion will focus on shifts >10 cm^−1^. The absorption peak representing the O-H stretching is affected by potential inter-molecular and/or intra-molecular hydrogen bonds [55]. The O–H peaks of pullulan shifted to a higher frequency upon electrospinning; specifically, a shift from 3296 cm^−1^, the peak corresponding to the pullulan standard, to 3308 cm^−1^ (δ 12 cm^−1^) and to 3327 (δ 30 cm^−1^), the peaks corresponding to 15%, and to 20 and 25% (w/v) pullulan fibers, respectively, was observed as pullulan was modified during electrospinning. Increasing frequencies of the O–H stretch indicate that hydrogen bonding within a network decreases [55]. This is accompanied by a shift in the C–O stretch to a higher frequency, consistent with decreasing hydrogen bonding upon electrospinning. As C–H bonds do not participate in hydrogen bonding, the small changes in the position of the C–H stretching peaks (all < 10 cm^−1^) are likely not significant. Therefore, these data show that electrospinning disrupts the hydrogen bonding between pullulan molecules. 

Figure 9B shows the FTIR spectra of pullulan electrospun fibers containing HP-β-CD. The spectra of pure ground components (pullulan, HP-β-CD) and their physical mixture were also included for comparison. An inset is included to highlight peak shifts for the O–H stretches; values for the O–H and C–O stretches are also summarized in Table S2. As intermolecular interactions occur between two molecules, the O–H region of an FTIR spectra shifts to a lower frequency. This has been previously observed when inter-molecular hydrogen bonds were formed between pullulan chains and other polymers, like chitosan [18] and gelatin [19] during electrospinning, as well as between HP-β-CD and quercetin to form an inclusion complex [56]. In the pullulan/ HP-β-CD electrospun fibers, we expect the OH groups along the pullulan backbone to form new hydrogen bonds with the OH groups on the HP-β-CD surface, creating physical crosslinks between the two molecules. Pullulan could also form hydrogen bonds with the OH groups within the HP-β-CD core as threading occurs, but as mentioned previously, we do not expect complexation of pullulan within HP-β-CD’s cavity. Given the structural similarities between pullulan and HP-β-CD, it is challenging to determine if a certain fraction of pullulan molecules in solution are in a more hydrophobic environment.

As observed in Figure 9B, the O–H band of HP-β-CD shifted from 3334 cm^−1^ to lower frequencies when it was electrospun with pullulan. This may be an indication of hydrogen bonding interactions occurring between HP-β-CD and pullulan in the spunfibers. Specifically, the O–H stretch of HP-β-CD shifted δ −24 cm^−1^ with respect to the 25% pullulan /10% HP-β-CD spunfiber. As the concentration of pullulan decreased in the 10% HP-β-CD spunfibers, the magnitude of this shift decreased, which indicates that higher concentrations of pullulan led to the formation of more hydrogen bonds between HP-β-CD and pullulan. Further insight can be obtained by comparing the spectra of HP-β-CD powder with the spunfibers containing different amounts of HP-β-CD. The increase in the magnitude of the hydroxyl group shift from δ −2 to −22 cm^−1^ when HP-β-CD is increased from 10 to 30% in the pullulan spunfiber suggests that hydrogen bonding is fully responsible for the decrease in frequency. As pullulan has a lower O–H frequency (3296 cm^−1^) than HP-β-CD (3334 cm^−1^), the frequency of the O–H peak would be expected to have a higher value if the HP-β-CD alcohols dominated the spectra. Therefore, in the electrospun fibers, hydrogen bonding appears to increase relative to the physical mixture of HP-β-CD and pullulan. However, these are still small changes in a signal spanning ~600 cm^−1^ that consists of several overlapping O–H stretching signals, and hence the amount of hydrogen bonds cannot be conclusively determined from these analyses. Shifts in the C–O stretches among the pullulan/ HP-β-CD electrospun fibers were small and not significant; despite the fact that they were shifted to higher frequencies than pullulan alone or the HP-β-CD-pullulan physical mixture, the O–H stretches are most important for determining hydrogen bonding that the C–O stretches.

## 4. Conclusions

We report for the first time that novel pullulan submicron fiber mats incorporating HP-β-CD were successfully produced via electrospinning from aqueous solutions. This is an important outcome since electrospinning in water would avoid the need for post-processing treatments and reduce the degradation of active ingredients to be encapsulated into the spunfibers. Our results showed that the entanglement concentration (C_E_) was the minimum concentration required for obtaining beaded spunfibers, whereas the optimal concentration required for effective electrospinning and the formation of continuous, well-formed and/or bead-free nanofibers was 1.59 to 3 times the C_E_, depending on the HP-β-CD concentration. The inclusion of 30% w/v HP-β-CD improved the electrospinnability of pullulan, promoting the formation of bead-free nanofibers at almost half the required polymer content (12 versus 20% w/v) in neat pullulan systems. DSC, DLS and FTIR analysis suggest that fiber morphology improvement is probably due to an association between pullulan and HP-β-CD that facilitated crosslinking of pullulan chains through the formation of inter-molecular hydrogen bonds. These findings are meaningful as the incorporation of HP-β-CD not only reduces the amount of pullulan to use but also adds value to pullulan spunfibers and widens their range of applications by increasing their encapsulation functionality without compromising their morphology and diameter. This study is the first step towards the use of pullulan/ HP-β-CD spunfibers as potential encapsulation matrices of unstable, poorly water-soluble active compounds for delivery applications (manuscript in preparation). 

## Figures and Tables

**Table 1:** Rheological properties of pure pullulan and pullulan/HP-β-CD solutions and morphology and average diameter of resulting spunfibers.

Solutions (Pullulan/HP-β-CD) (% w/v)	k (mPa.s ^n^)	n	Fiber Morphology	Average Fiber Diameter, ADF (nm)
0.5/0	0.81 ± 0.0006 ^a^	1.10 ± 0.00034 ^a^	No fiber formation	-
1/0	1.88 ± 0.07 ^b^	1.05 ± 0.0047 ^b^	No fiber formation	-
2/0	3.95 ± 0.18 ^c^	1.02 ± 0.0040 ^b^	No fiber formation	-
5/0	22.4 ± 2.7 ^e^	1.001 ± 0.0055 ^b^	Beaded structures	-
8/0	67.5 ± 11 ^f^	0.978 ± 0.0084 ^c^	Fibers with many beads	-
10/0	173 ± 19 ^g^	0.943 ± 0.0086 ^e^	Fibers with many beads	-
12/0	483 ± 83 ^h^	0.895 ± 0.0093 ^g^	Fibers with bead structures	-
15/0	1743 ± 402 ^i^	0.825 ± 0.012 ^i^	Fibers with bead structures	-
20/0	6170 ± 866 ^j^	0.754 ± 0.0089 ^k^	Bead-free fibers	886 ± 143 ^a^
25/0	16500 ± 50 ^l^	0.692 ± 0.0027 ^l^	Bead-free fibers	863 ± 87 ^a^
0.5/10	1.73 ± 0. 23 ^b^	1.04 ± 0.027 ^b^	No fiber formation	-
1/10	2.11 ± 0.02 ^b^	1.04 ± 0.00021 ^b^	No fiber formation	-
2/10	4.75 ± 0.11 ^c^	1.01 ± 0.0026 ^b^	No fiber formation	-
5/10	22.4 ± 1.55 ^e^	1.01 ± 0.00010 ^b^	Bead structures with few fibers	-
8/10	86.6 ± 6.39 ^f^	0.985 ± 0.00264 ^c^	Fibers with many beads	-
10/10	257 ± 44 ^g^	0.930 ± 0.0113 ^f^	Fibers with many beads	-
12/10	558 ± 76 ^h^	0.893 ± 0.00713 ^g^	Fibers with few beads	-
15/10	1710 ± 120 ^i^	0.831 ± 0.00298 ^i^	Fibers with few beads	-
20/10	8925 ± 61 ^j^	0.746 ± 0.0023 ^k^	Bead-free fibers	853 ± 130 ^a^
25/10	20179 ± 1171 ^m^	0.643 ± 0.016 ^m^	Bead-free fibers	823 ± 91 ^a^
0.5/30	3.66 ± 0.65 ^c^	1.01 ± 0.018 ^b^	No fiber formation	-
1/30	7.07 ± 0.31 ^d^	1.01 ± 0.0007 ^b^	No fiber formation	-
2/30	8.86 ± 0.08 ^d^	1.01 ± 0.00094 ^b^	No fiber formation	-
5/30	36.5 ± 0.96 ^e^	1.01 ± 0.00052 ^b^	Fibers with many beads	-
8/30	136 ± 7.61 ^g^	0.967 ± 0.00103 ^d^	Fibers with few beads	-
10/30	296 ± 17.5 ^g, h^	0.929 ± 0.00078 ^f^	Fibers with few beads	-
12/30	1380 ± 96 ^i^	0.852 ± 0.00502 ^h^	Bead-free fibers	866 ± 137 ^a^
15/30	3753 ± 147 ^k^	0.794 ± 0.0023 ^j^	Bead-free fibers	886 ± 225 ^a^
20/30	16392 ± 2441 ^l^	0.695 ± 0.015 ^l^	Bead-free fibers	882 ± 322 ^a^
25/30	44573 ± 9531 ^n^	0.617 ± 0.0108 ^n^	Bead-free fibers	1019 ± 200 ^a^

Rows with different letters are significantly different (*p* ≤ 0.05). k: consistency index; n: flow behavior index. Average fiber diameters are only given for smooth and bead-free spunfibers.

## Supplementary Materials

The following are available online at https://www.mdpi.com/2073-4360/12/11/2558/s1, Figure S1: SEM image of 150% (w/v) HP-β-CD; Figure S2: Dependence of electrospun fiber average diameter on pullulan concentration (% w/v) at different HP-β-CD levels; Figure S3: Size distributions of solutions of pullulan 20% w/v, HP-β-CD 30% w/v and pullulan 20%/HP-β-CD 30% w/v mixture measured via DLS at 25°C; Table S1: Electrical conductivities (mS/cm) of blends of pullulan/HP-B-CD at different concentrations; Table S2: FTIR peak values corresponding to O–H and C–O stretches.
