# Supplementary material for: Novel Electrospun Pullulan Fibers Incorporating Hydroxypropyl-β-Cyclodextrin: Morphology and Relation with Rheological Properties
Source: Polymers (Basel). 2020 Oct 31;12(11):2558. doi: 10.3390/polym12112558 (PMC7693914; doi:10.3390/polym12112558)
Supplement: Supplementary file 1 [file polymers-12-02558-s001.pdf]

Supplementary Materials

Figures and Tables, Poudel et al.

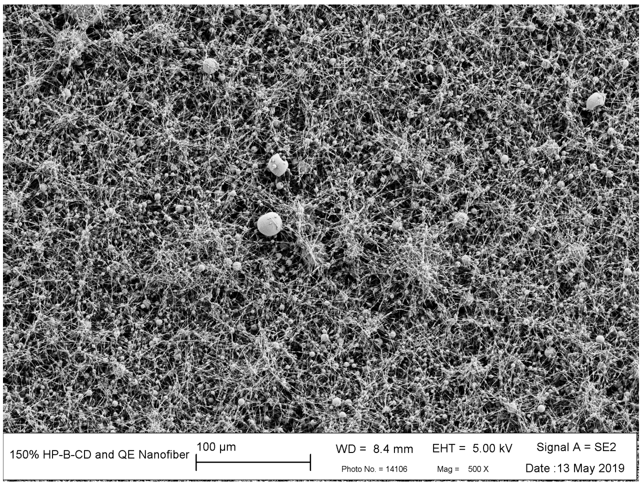

Figure S1. SEM image of 150% (w/v) HP-β-CD.

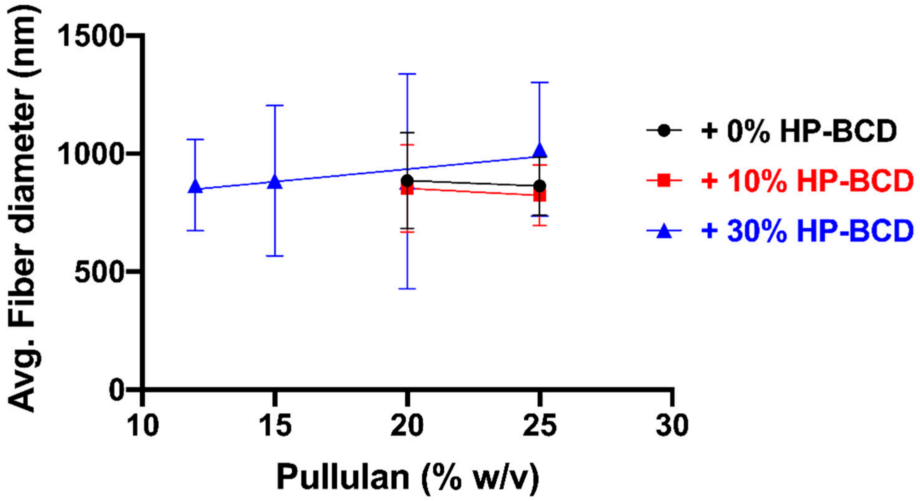

Figure S2. Dependence of electrospun fiber average diameter on pullulan concentration (% w/v) at different HP-β-CD levels

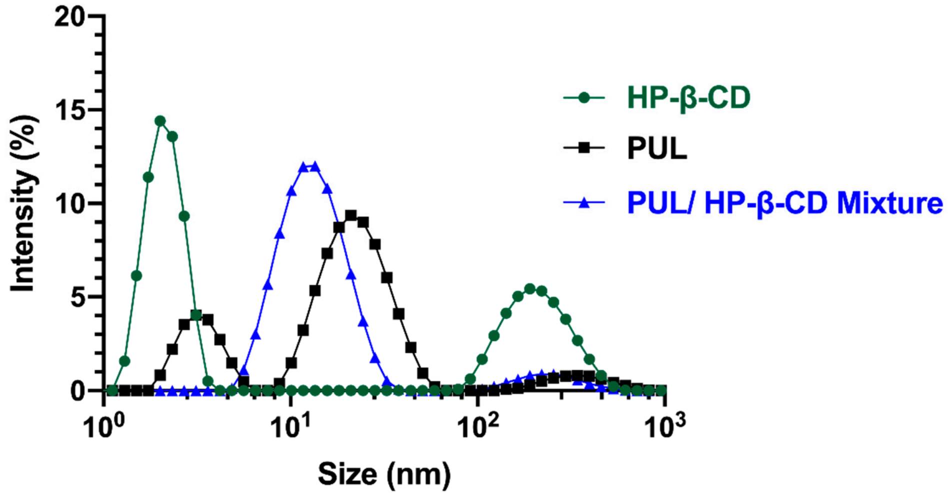

Figure S3. Size distributions of solutions of pullulan 20 % w/v, HP-β-CD 30% w/v and pullulan 20%/ HP-β-CD 30% w/v mixture measured via DLS at 25 °C

Table S1. Electrical conductivities (mS/cm) of blends of pullulan/HP-B-CD at different concentrations.

Pullulan Concentration	0% HP-*β*-CD	10% HP-*β*-CD	30% HP-*β*-CD
0.5 %w/v	0.00	0.00	0.10
1 %w/v	0.00	0.00	0.10
2 %w/v	0.00	0.00	0.10
5 %w/v	0.00	0.00	0.10
8 %w/v	0.00	0.00	0.10
10 %w/v	0.10	0.10	0.10
12 %w/v	0.10	0.10	0.10
15 %w/v	0.10	0.10	0.10
20 %w/v	0.10	0.10	0.10
25 %w/v	0.10	0.10	0.10

Table S2. FTIR peak values corresponding to O–H and C–O stretches

Entry	Sample	O–H stretch (cm^{-1})	C–O stretch (cm^{-1})
1	15% Pullulan 10% HPBCD	3332	1024
2	20% Pullulan 10% HPBCD	3330	1024
3	25% Pullulan 10% HPBCD	3310	1023
4	15% Pullulan 30% HPBCD	3312	1019
5	1:1 Physical Mixture	3319	1000
6	HPBCD	3334	1022
7	Pullulan	3296	993
8	15% Pullulan	3308	1020
9	20% Pullulan	3327	1020
10	25% Pullulan	3327	1019
